# Supplementary material for: Structural insights into PA3488-mediated inactivation of Pseudomonas aeruginosa PldA
Source: Nat Commun. 2022 Oct 10;13:5979. doi: 10.1038/s41467-022-33690-2 (PMC9550806; doi:10.1038/s41467-022-33690-2)
Supplement: Supplementary file 1 — Supplementary Information [file 41467_2022_33690_MOESM1_ESM.pdf]

Supplementary Information

Structural insights into PA3488-mediated inactivation of *Pseudomonas aeruginosa* PldA

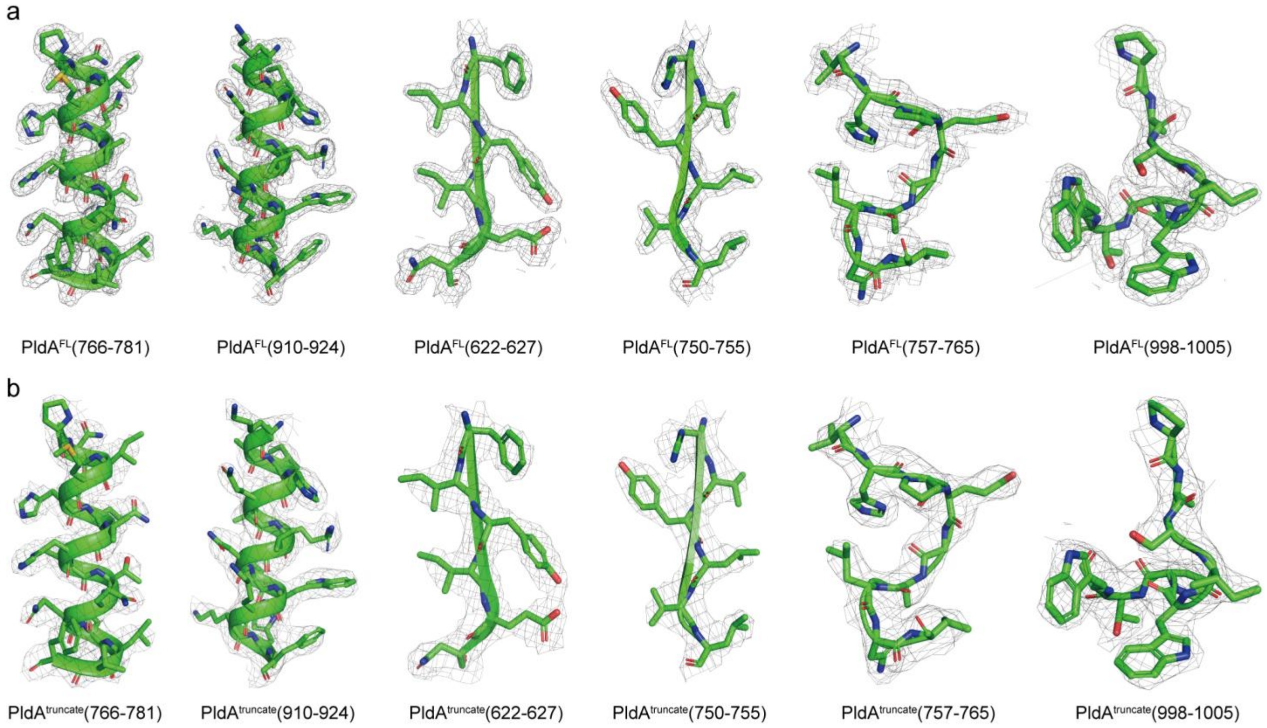

Supplementary Figure 1 Representative density from PldA^{FL} and PldA^{truncate}. a-b Representative density of α helices, β sheets and loops from PldA^{FL} (**a**) and PldA^{truncate} (**b**), respectively. Fo-Fc omit map at 1σ.

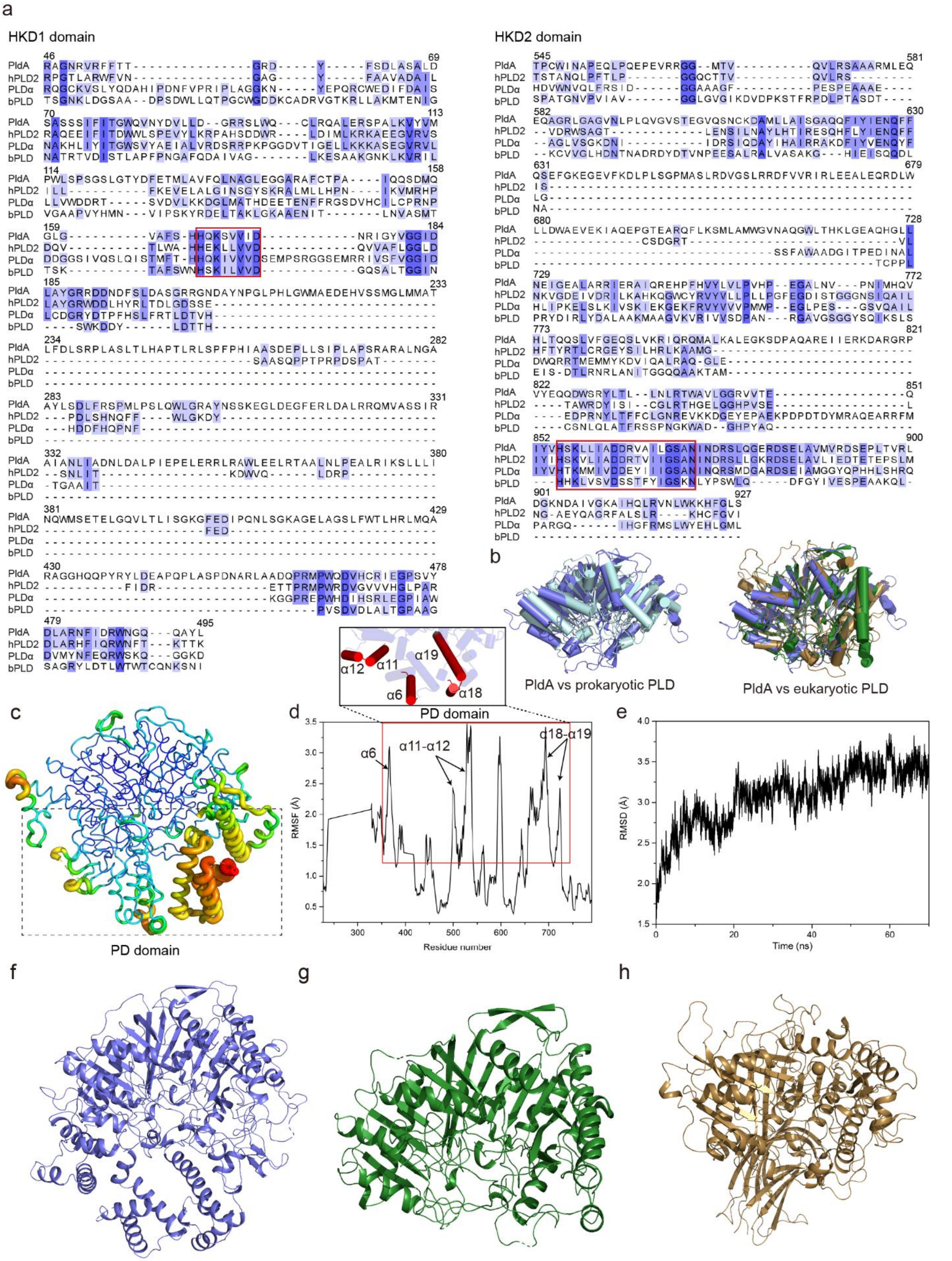

Supplementary Figure 2 Conservation and stability of PldA. a Sequence alignment of PldA, human PLD2 (hPLD2), plant PLD (PLDα), and bacterial PLD (bPLD). The sequence alignment was generated with Muscle and visualized using Jalview. The active site residues are underlined.

b Structural comparisons of CCD of PldA with prokaryotic PLD (left panel, RMSD of 10.753 for 300 matched Cα pairs) and eukaryotic PLDs (right panel, RMSD of 0.770 for 278 matched Cα pairs of human and RMSD of 0.966 for 272 matched Cα pairs of plant). *Streptomyces sp.* (palecyan, PDB:1FOI), *Homo sapiens* (forest, PDB:6OHO) and *A. thalian*a (sand, PDB:6KZ9). **c-e** Stability of PldA. B-factor putty representation of $PldA^{FL}$, Stability: blue > yellow > red (**c**). The root-mean-square fluctuation (RMSF) of $PldA^{FL}$ during the simulation was calculated with reference to an energy-minimized initial crystal structure. The red box highlights the PD region possessing high RMSF (**d**). Representative root-mean-square deviation of PldA Cα in the molecular dynamic trajectory (**e**). The results indicate that the PD domain is more flexible than the HKD domains. **f-h** Structures of PLDs from different species including PldA (**f**), hPLD2 (**g**) and PLDα (**h**).

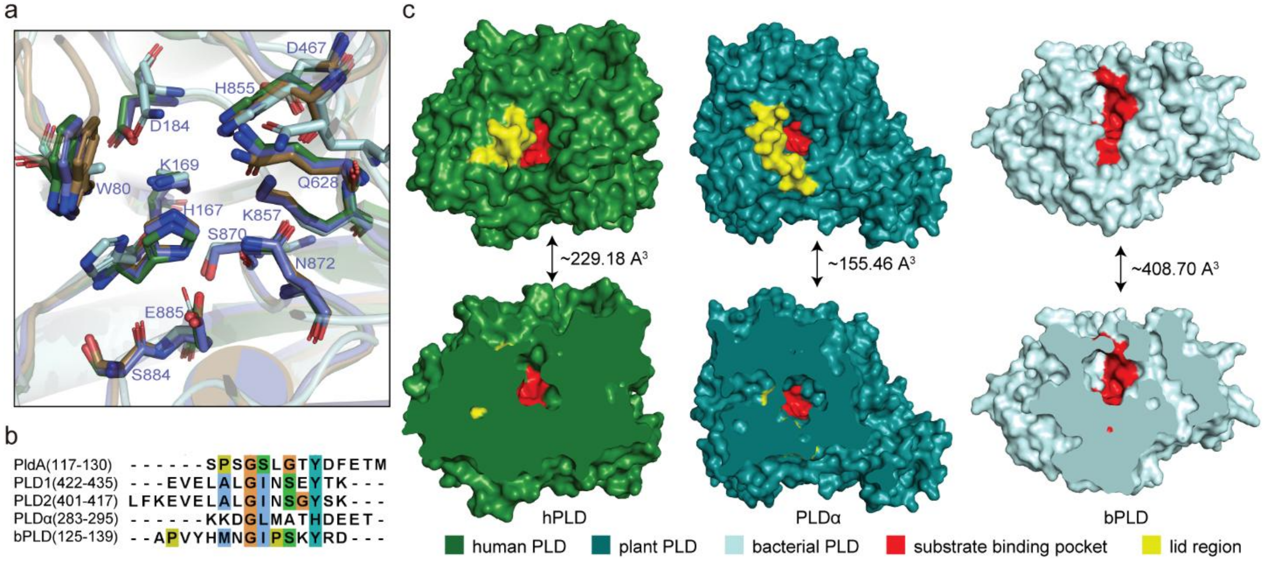

Supplementary Figure 3 Comparison of active pockets from PLDs. a Alignment of conserved active sites in the HKD domain from PLDs (PldA, hPLD2, and PLDα are colored as in Fig. 1C, bPLD is colored pale cyan). **b** Sequence alignment of lid region from different species. **c** Surface (upper) and cross-section (bottom) representation of active pockets from PLDs. The volume of each pocket in PLDs is calculated through a web server-CASTp 3.0. The significant conformational change of the lid region is highlighted in yellow and the active site is highlighted in red.

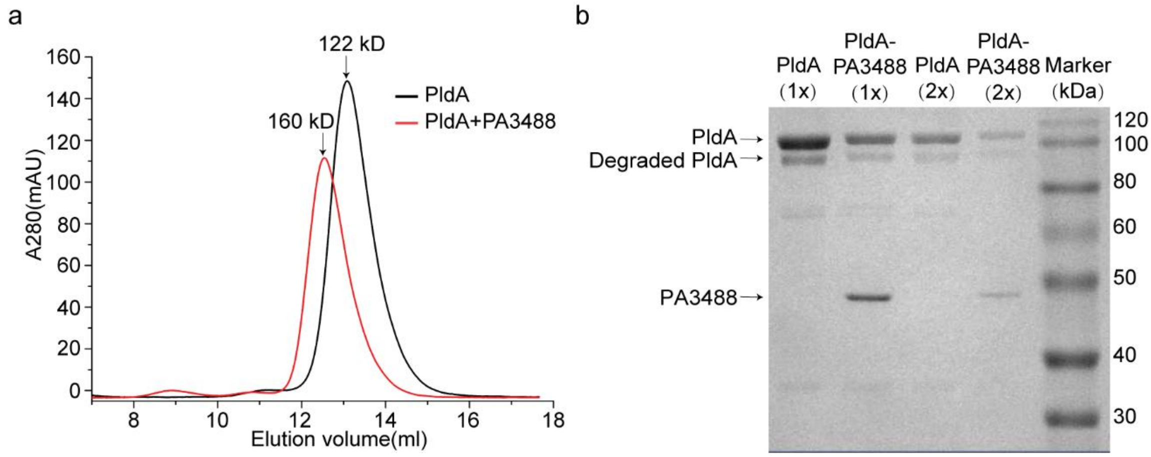

Supplementary Figure 4 Protein purification of PldA and PldA-PA3488 complex. a Size exclusion chromatography of PldA and PldA-PA3488 complex. **b** SDS-PAGE gel of peak fractions collected in the size exclusion chromatography. Samples of PldA and PldA-PA3488 were loaded repeatedly by diluting to 2-fold. Source data are provided as a Source Data file.

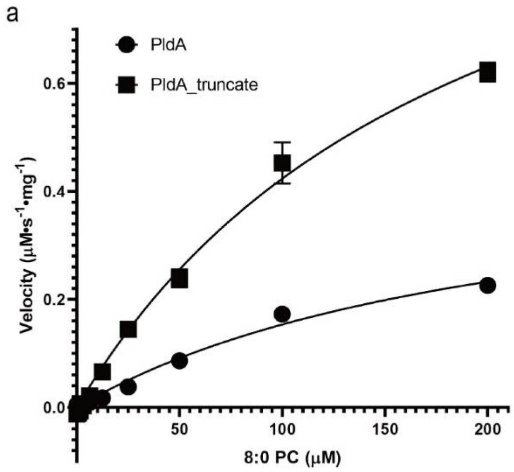

b

Kinetic parameter	PldA	$PldA^{truncate}$
K_m (μM)	**214.3**	**192.1**
k_{cat} (s^{-1})	**64.17**	**154.6**
V_{max} (μmol•s^{-1}•mg^{-1})	**0.4825**	**1.237**
k_{cat}/K_m ($μM^{-1}$•s^{-1})	**0.30**	**0.80**

Supplementary Figure 5 ***In vitro*** **kinetic assay of PldA and truncated PldA using di8:0-PC as substrate. a** Michaelis-Menten kinetics of PldA and $PldA^{truncate}$. Results are means ± SD, n = 3 biologically independent samples. Dots indicate the corresponding data distribution. Source data are provided as a Source Data file. **b** The kinetic parameters were calculated and shown in the table.

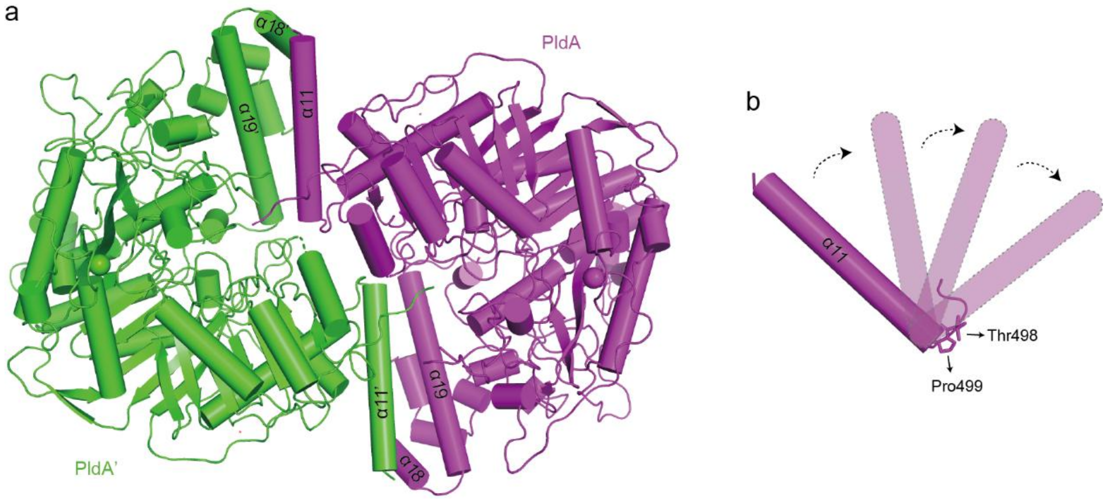

Supplementary Figure 6 α11 stabilizes the structure of PldA^{truncate}. a The flipped α11 is stabilized by the α18' and α19' from a symmetric PldA' in the unit cell. **b** α11 anchors on the Thr^{498} and Pro^{499} to make it flip flexibly.

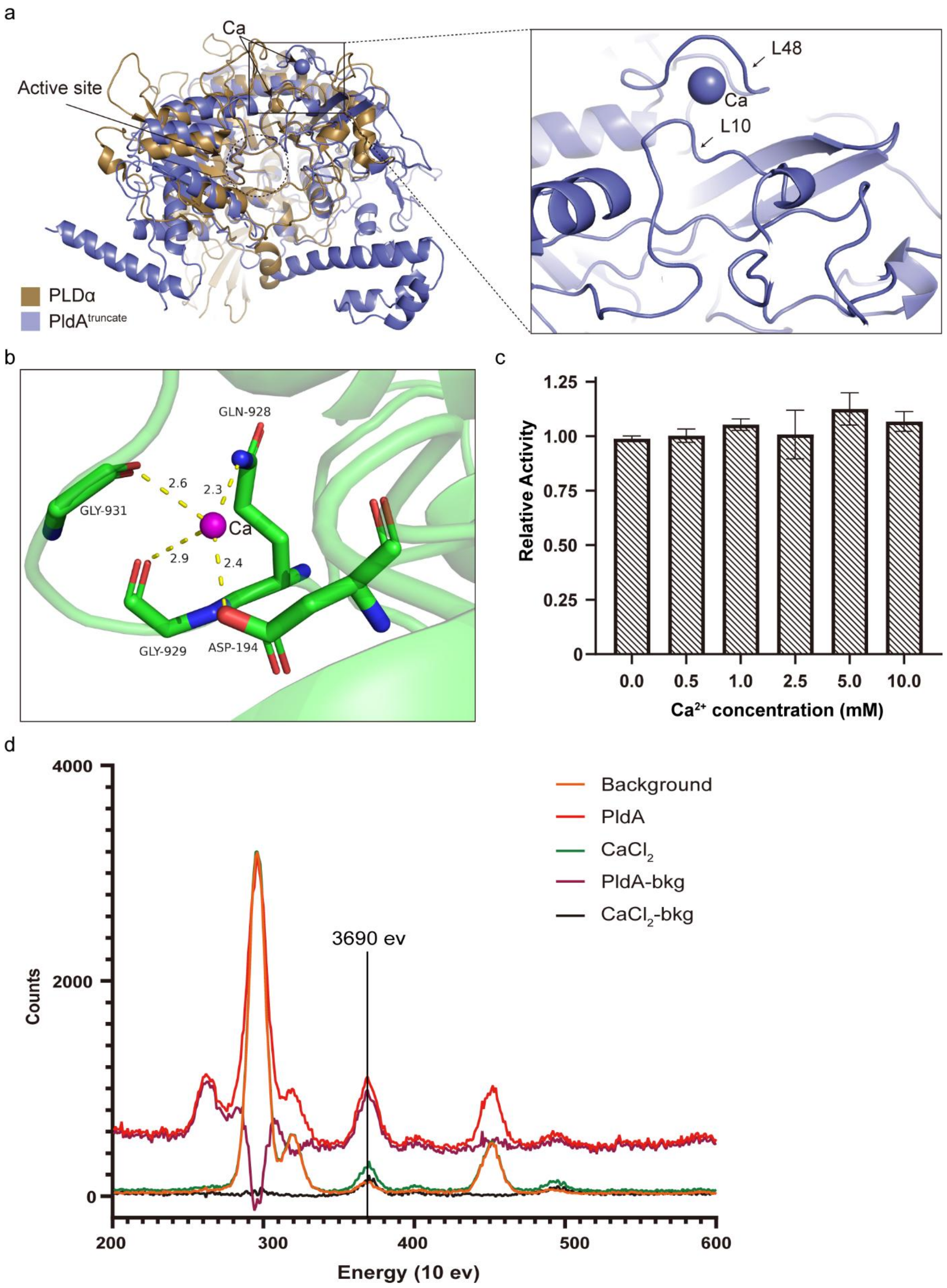

Supplementary Figure 7 Ca^{2+} in the truncated PldA structure. a Comparison of the Ca^{2+} position in the PldA and Pldα. The results showed Ca^{2+} in the PldA is further away from the active site. **b** The Ca^{2+} binding site in the $PldA^{FL}$ structure. Four residues binding with Ca^{2+} and their distances are labeled. **c** PldA enzyme activity measured with different concentrations of Ca^{2+}. Results are means ± SD, n = 3 biologically independent samples. Source data are provided as a Source Data file. **d** μ-XRF analysis of PldA. The purple peak in 3690 ev indicates the existence of Ca.

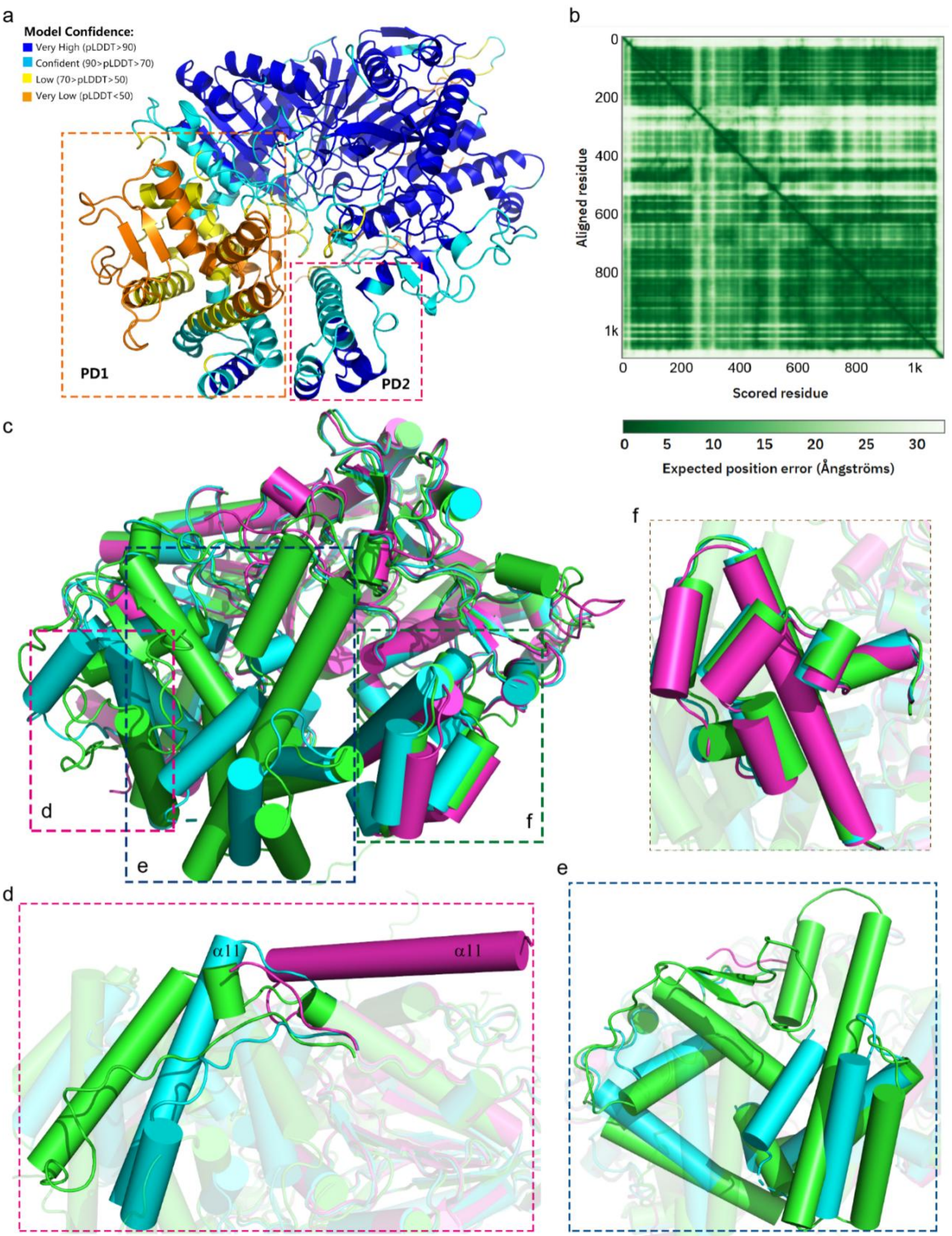

Supplementary Figure 8 AlphaFold2 prediction structure of PldA. a Overall structure of AlphaFold2 predicted PldA in cartoon representation. Different colors indicate different pLDDT values and the two PD domains are marked. **b** The PAE value of predicted PldA structure. **c** Superposition of AlphaFold2 predicted PldA (green), PldA^{FL}(cyan) and PldA^{truncate}. **d-f** Detailed comparison of different domains: The linker domain (**d**), PD1 domain (**e**) and PD2 domain (**f**).

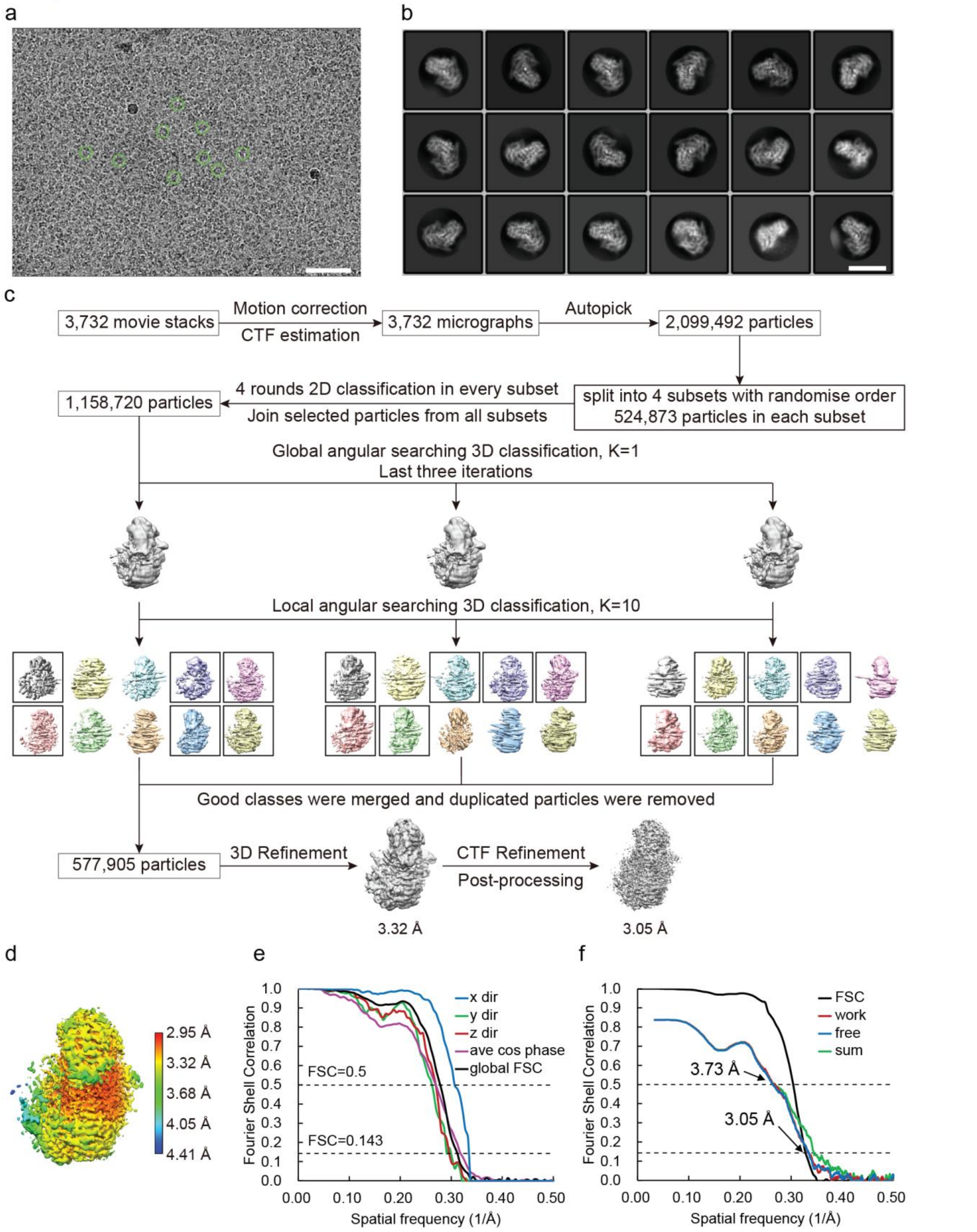

Supplementary Figure 9 Cryo-EM data analysis. Cryo-EM reconstruction of PldA–PA3488 complex. **a** A representative motion-corrected cryo-EM micrograph from 3732 micrographs with typical particles marked by green circle. Scale bar, 50 nm. **b** Typical good, reference-free 2D class averages. Scale bar, 10 nm. **c** Flowchart of single-particle processing. Details can be seen in the "Image processing" section. **d** Local resolution of the final reconstruction colored based on the provided scale. **e** Estimation of the directional FSCs, with the *x* direction (blue), *y* direction (green), and *z* direction (red). The global FSC is represented in black. The sphericity coefficient is

0.945. **f** Post-processing and model cross-validation FSCs. FSC curve of post-processing (black) and FSC curves of the refined model versus the overall map that it was refined against (sum, green), of the model refined against the first half map versus that same map (work, red), and of the model refined against the first half map versus the second map (free, red). The small difference between the red and blue curves indicates that the refinement of the atomic coordinates was not affected by overfitting.

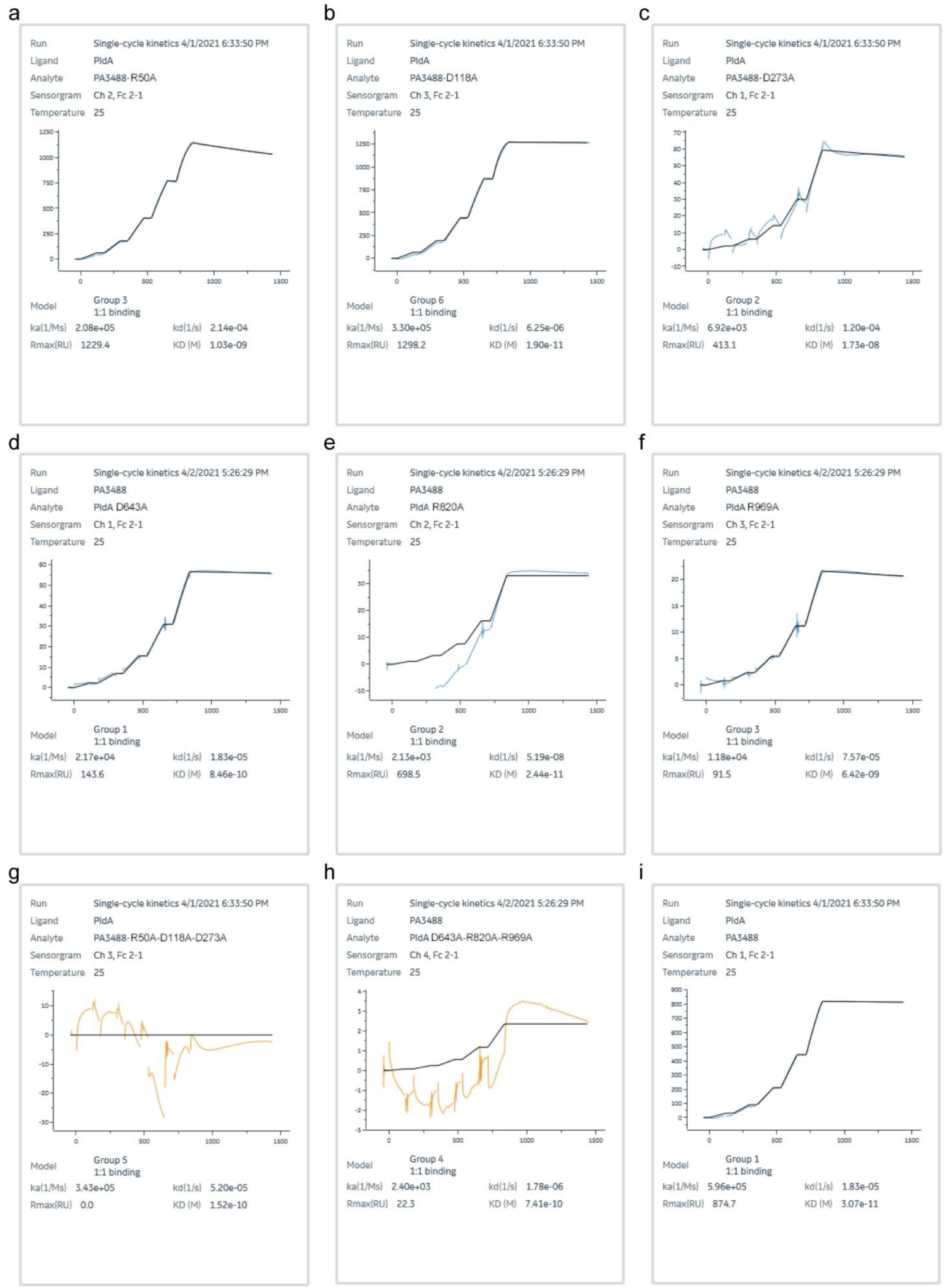

Supplementary Figure 10 Kinetics and affinity constants for wild-type and mutant PldA-PA3488 complex. These data correspond to Table 1.

Supplementary Table 1 Data collection, phasing and refinement statistics

	$PldA^{FL}$ (PDB 7V53)	$PldA^{truncate}$ (PDB 7V55)
**Data collection**		
Space group	$P2_12_12_1$	$P3_121$
Unit cell		
a, b, c (Å)	62.33, 111.47, 144.76	83.57 83.57 242.34
α, β, γ (°)	90, 90, 90	90, 90, 120
Resolution (Å)	88.32-2.10 (2.15-2.10)	72.37-3.00 (3.18-3.00)
Completeness (%)	96.2 (99.5)	94.4 (99.3)
Multiplicity	6.5 (6.3)	5.3 (5.6)
$CC_{1/2}$	0.997 (0.930)	0.996 (0.746)
R_{merge}	0.079 (0.389)	0.110 (0.663)
I/σI	13.7 (4.0)	11.8 (2.2)
**Refinement**		
No. reflections	57623	19226
R_{work}/R_{free} (%)	17.7/21.5	18.9/24.9
No. atoms		
Protein	7162	6118
Ligand	\	1 (Ca^{2+})
Water	536	3
B factors (Å)		
Protein	35.08	60.73
Ligand (ligand species)	\	72.99
Water	36.19	47.3
R.m.s deviationss		
Bond lengths (Å)	0.007	0.009
Bond angles (°)	0.843	1.187
Ramachandran		
Favored (%)	92.2	86.4
Allowed (%)	7.5	13.1

[a]Values in parentheses are for the highest-resolution shell

Supplementary Table 2 Cryo-EM data collection and model statistics.

**Data collection and processing**	
Magnification	130,000
Voltage (kV)	300
camera	K3
Electron exposure (e–/Å2)	50
Defocus range (μm)	-1.5 ~ -2.5
Pixel size (Å)	0.668
Micrographs (no.)	3,732
Initial particle images (no.)	2,099,492
Final particle images (no.)	577,905
Symmetry imposed	C1
Map resolution (Å)	3.05
Map sharpening B factor (Å2)	-130
FSC threshold	0.143
Map resolution range (Å)	4.41-2.95
EMDB number	EMD-32438
**Refinement**	
Initial model used	generated in RELION (v3.1)
Model composition	
Non-hydrogen atoms	94,39
Protein residues	1,215
R.m.s. deviations	
Bond lengths (Å)	0.004
Bond angles (°)	0.712
Validation	
MolProbity score	2.10
Clashscore	12.88
Rotamer outliers (%)	0
Cβ outliers (%)	0
Ramachandran plot	
Favored (%)	92.06
Allowed (%)	7.94
Disallowed (%)	0
PDB accession number	7WDK
