# Supplementary material for: Structural insights into PA3488-mediated inactivation of Pseudomonas aeruginosa PldA
Source: Nat Commun. 2022 Oct 10;13:5979. doi: 10.1038/s41467-022-33690-2 (PMC9550806; doi:10.1038/s41467-022-33690-2)
Supplement: Supplementary file 3 — Reporting Summary [file 41467_2022_33690_MOESM3_ESM.pdf]

nature portfolio

Corresponding author(s): Sen-fang Sui, Yuhui Dong and Yanhua Li

Last updated by author(s): Sep 6, 2022

Reporting Summary

Nature Portfolio wishes to improve the reproducibility of the work that we publish. This form provides structure for consistency and transparency in reporting. For further information on Nature Portfolio policies, see our Editorial Policies and the Editorial Policy Checklist.

Statistics

For all statistical analyses, confirm that the following items are present in the figure legend, table legend, main text, or Methods section.

n/a	Confirmed	
☐	☒	The exact sample size (*n*) for each experimental group/condition, given as a discrete number and unit of measurement
☐	☒	A statement on whether measurements were taken from distinct samples or whether the same sample was measured repeatedly
☐	☒	The statistical test(s) used AND whether they are one- or two-sided<br>*Only common tests should be described solely by name; describe more complex techniques in the Methods section.*
☐	☒	A description of all covariates tested
☐	☒	A description of any assumptions or corrections, such as tests of normality and adjustment for multiple comparisons
☐	☒	A full description of the statistical parameters including central tendency (e.g. means) or other basic estimates (e.g. regression coefficient) AND variation (e.g. standard deviation) or associated estimates of uncertainty (e.g. confidence intervals)
☒	☐	For null hypothesis testing, the test statistic (e.g. *F*, *t*, *r*) with confidence intervals, effect sizes, degrees of freedom and *P* value noted<br>*Give P values as exact values whenever suitable.*
☒	☐	For Bayesian analysis, information on the choice of priors and Markov chain Monte Carlo settings
☒	☐	For hierarchical and complex designs, identification of the appropriate level for tests and full reporting of outcomes
☒	☐	Estimates of effect sizes (e.g. Cohen's *d*, Pearson's *r*), indicating how they were calculated

Our web collection on statistics for biologists contains articles on many of the points above.

Software and code

Policy information about availability of computer code

Data collection	Serial EM was used for cryo-EM data collection; BL19U1 and BL17U beamlines were used for x-ray crystallography data collection.
Data analysis	MotionCor2, CTFFIND-4.10, RELION 3.1.2, Chimera v.1.14, ChimeraX v.1.2, Coot 0.8.9.2, Phenix 1.19.1-4122, pymol v2.x, XDS, PHASER, AutoDock 4, GROMACS 2021.3, GraphPad Prism 5, PyMca 5.7.5, Origin 8.0.

For manuscripts utilizing custom algorithms or software that are central to the research but not yet described in published literature, software must be made available to editors and reviewers. We strongly encourage code deposition in a community repository (e.g. GitHub). See the Nature Portfolio guidelines for submitting code & software for further information.

Data

Policy information about availability of data

All manuscripts must include a data availability statement. This statement should provide the following information, where applicable:

- Accession codes, unique identifiers, or web links for publicly available datasets
- A description of any restrictions on data availability
- For clinical datasets or third party data, please ensure that the statement adheres to our policy

The coordinates and structure factors for PldAFL and PldAtruncate have been deposited in the Protein Data Bank with accession codes 7V53 [https://doi.org/10.2210/pdb7V53/pdb] and 7V55 [https://doi.org/10.2210/pdb7V55/pdb], respectively. The cryo-EM 3D maps of the PldA-PA3488 complex have been deposited in the Electron Microscopy Data Bank (EMDB) with accession code EMD-32438 . Atomic coordinates of PldA-PA3488 complex (PDB: 7WDK) [https://doi.org/10.2210/pdb7WDK/pdb] have been deposited in the Protein Data Bank. All data needed to evaluate the conclusions in the paper are shown in the article and/or the supplementary materials. Source data are provided with this paper.

Field-specific reporting

Please select the one below that is the best fit for your research. If you are not sure, read the appropriate sections before making your selection.

[x] Life sciences   [ ] Behavioural & social sciences   [ ] Ecological, evolutionary & environmental sciences

For a reference copy of the document with all sections, see nature.com/documents/nr-reporting-summary-flat.pdf

Life sciences study design

All studies must disclose on these points even when the disclosure is negative.

Sample size	For the full-length PldA, truncated PldA and PldA-PA3488 complex, the crystal and cryo-EM datasets were collected and generated interpretable map with sufficient resolution reported in this study respectively. Due to the resolution is high (2.1-3.05 angstroms), we judged the sample size is enough. The numbers of reflections and particles used in this study are summarized in supplementary Fig. 1 and supplementary Fig. 2.
Data exclusions	For cryo-EM data set of PldA-PA3488 complex, the images and particles can not generate good reconstructions are excluded, according to the Methods section in this work. There is no data exclusion in other experiments.
Replication	The cryo-EM data set of PldA-PA3488 complex that contains millions particles of PldA-PA3488 complex has inherent replication. Crystals of proteins,SDS-PAGE....experiments were repeated independently at least three times with similar results.
Randomization	Randomization is not relevant to this study. Because the data analysis is automatically processed in the cryo-EM data processing package RELION-3.1.2 and the crystal data processing package XDS based on the particle shapes, the particle sizes, the quality of diffraction, etc.
Blinding	Not applicable in this study, because the exact sample under investigation needed to be known.

Reporting for specific materials, systems and methods

We require information from authors about some types of materials, experimental systems and methods used in many studies. Here, indicate whether each material, system or method listed is relevant to your study. If you are not sure if a list item applies to your research, read the appropriate section before selecting a response.

Materials & experimental systems

n/a	Involved in the study	
[x]	[ ]	Antibodies
[x]	[ ]	Eukaryotic cell lines
[x]	[ ]	Palaeontology and archaeology
[x]	[ ]	Animals and other organisms
[x]	[ ]	Human research participants
[x]	[ ]	Clinical data
[x]	[ ]	Dual use research of concern

Methods

n/a	Involved in the study	
[x]	[ ]	ChIP-seq
[x]	[ ]	Flow cytometry
[x]	[ ]	MRI-based neuroimaging
