# Supplementary material for: Structural insights into PA3488-mediated inactivation of Pseudomonas aeruginosa PldA
Source: Nat Commun. 2022 Oct 10;13:5979. doi: 10.1038/s41467-022-33690-2 (PMC9550806; doi:10.1038/s41467-022-33690-2)
Supplement: Supplementary file 4 — Source Data [file 41467_2022_33690_MOESM4_ESM.zip › TOF sourcedata/2022-06-28-YY0126/Concise Summary Report (20).htm]

Concise Summary Report (20)


# Mascot Search Results

```
User            : niulili
Email           : niulili@ibp.ac.cn
Search title    : 20
MS data file    : 20.mgf
Database        : liyanhua20220628 20220628 (247 sequences; 128998 residues)
Timestamp       : 1 Jul 2022 at 03:44:24 GMT
Top Score       : 130 for LYH,
```

### Mascot Score Histogram

Protein score is -10\*Log(P), where P is the
probability that the observed match is a random event.  
Protein scores greater
than 36 are significant (p<0.05).  

### Concise Protein Summary Report

|  |  |  |
| --- | --- | --- |
|  | Protein Summary Concise Protein Summary Export Search Results | Help |
|  | Significance threshold p< | Max. number of hits |
|  | Preferred taxonomy All entries . . Archaea (Archaeobacteria) . . Eukaryota (eucaryotes) . . . . Alveolata (alveolates) . . . . . . Plasmodium falciparum (malaria parasite) . . . . . . Other Alveolata . . . . Metazoa (Animals) . . . . . . Caenorhabditis elegans . . . . . . Drosophila (fruit flies) . . . . . . Chordata (vertebrates and relatives) . . . . . . . . bony vertebrates . . . . . . . . . . lobe-finned fish and tetrapod clade . . . . . . . . . . . . Mammalia (mammals) . . . . . . . . . . . . . . Primates . . . . . . . . . . . . . . . . Homo sapiens (human) . . . . . . . . . . . . . . . . Other primates . . . . . . . . . . . . . . Rodentia (Rodents) . . . . . . . . . . . . . . . . Mus. . . . . . . . . . . . . . . . . . . Mus musculus (house mouse) . . . . . . . . . . . . . . . . Rattus . . . . . . . . . . . . . . . . Other rodentia . . . . . . . . . . . . . . Other mammalia . . . . . . . . . . . . Xenopus laevis (African clawed frog) . . . . . . . . . . . . Other lobe-finned fish and tetrapod clade . . . . . . . . . . Actinopterygii (ray-finned fishes) . . . . . . . . . . . . Takifugu rubripes (Japanese Pufferfish) . . . . . . . . . . . . Danio rerio (zebra fish) . . . . . . . . . . . . Other Actinopterygii . . . . . . . . Other Chordata . . . . . . Other Metazoa . . . . Dictyostelium discoideum . . . . Fungi . . . . . . Saccharomyces Cerevisiae (baker's yeast) . . . . . . Schizosaccharomyces pombe (fission yeast) . . . . . . Pneumocystis carinii . . . . . . Other Fungi . . . . Viridiplantae (Green Plants) . . . . . . Arabidopsis thaliana (thale cress) . . . . . . Oryza sativa (rice) . . . . . . Other green plants . . . . Other Eukaryota . . Bacteria (Eubacteria) . . . . Actinobacteria (class) . . . . . . Mycobacterium tuberculosis complex . . . . . . Other Actinobacteria (class) . . . . Firmicutes (gram-positive bacteria) . . . . . . Bacillus subtilis . . . . . . Mycoplasma . . . . . . Streptococcus Pneumoniae . . . . . . Streptomyces coelicolor . . . . . . Other Firmicutes . . . . Proteobacteria (purple bacteria) . . . . . . Agrobacterium tumefaciens . . . . . . Campylobacter jejuni . . . . . . Escherichia coli . . . . . . Neisseria meningitidis . . . . . . Salmonella . . . . . . Other Proteobacteria . . . . Other Bacteria . . Viruses . . . . Hepatitis C virus . . . . Other viruses . . Other (includes plasmids and artificial sequences) . . unclassified . . Species information unavailable | |


 


 
 

 
 
 
 
 

 
 


   

|  |  |
| --- | --- |
| **1.** | LYH    **Mass:** 122664   **Score:** **130**  **Expect:** 2.5e-11  **Matches:** 27 |

|  |  |
| --- | --- |
|  | Q862S4TREMBL:Q862S4(Bostaurus)Similartoproalpha1(I)collagen(Fragment)    **Mass:** 13303    **Score:** 14     **Expect:** 11  **Matches:** 2 |

|  |  |
| --- | --- |
|  | P02768-1SWISS-PROT:P02768-1Tax\_Id=9606Gene\_Symbol=ALBIsoform1ofSerumalbuminprecursor    **Mass:** 71317    **Score:** 12     **Expect:** 14  **Matches:** 4 |

---

|  |  |
| --- | --- |
| **2.** | P41361SWISS-PROT:P41361(Bostaurus)Antithrombin-IIIprecursor    **Mass:** 52827    **Score:** 36     **Expect:** 0.063  **Matches:** 11 |

---

### Search Parameters

```
Type of search         : Peptide Mass Fingerprint
Enzyme                 : Trypsin
Fixed modifications    : Carbamidomethyl (C)
Variable modifications : Oxidation (M)
Mass values            : Monoisotopic
Protein Mass           : Unrestricted
Peptide Mass Tolerance : ± 100 ppm
Peptide Charge State   : 1+
Max Missed Cleavages   : 2
Number of queries      : 85
Selected for scoring   : 45
```

|  |
| --- |
| **Mascot:** http://www.matrixscience.com/ |
