# Supplementary figures and images for: Structural insights into PA3488-mediated inactivation of Pseudomonas aeruginosa PldA
Source: Nat Commun. 2022 Oct 10;13:5979. doi: 10.1038/s41467-022-33690-2 (PMC9550806; doi:10.1038/s41467-022-33690-2)

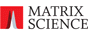

Supplement: Supplementary file 4 — Source Data [file 41467_2022_33690_MOESM4_ESM.zip › TOF sourcedata/2022-06-28-YY0126/Concise Summary Report (20)_files/88x31_logo_white.gif]

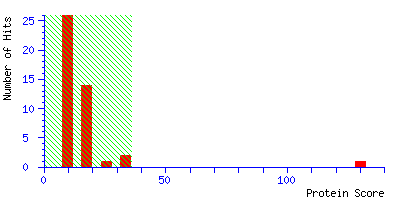

Supplement: Supplementary file 4 — Source Data [file 41467_2022_33690_MOESM4_ESM.zip › TOF sourcedata/2022-06-28-YY0126/Concise Summary Report (20)_files/score_gif.gif]

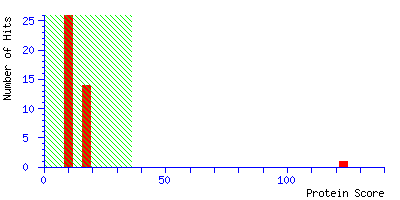

Supplement: Supplementary file 4 — Source Data [file 41467_2022_33690_MOESM4_ESM.zip › TOF sourcedata/2022-06-28-YY0126/Concise Summary Report (40)_files/score_gif2IXQDMHQ.gif]

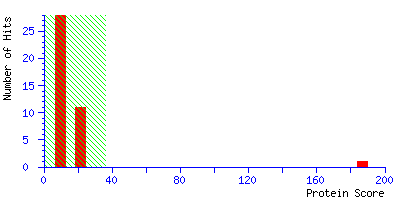

Supplement: Supplementary file 4 — Source Data [file 41467_2022_33690_MOESM4_ESM.zip › TOF sourcedata/2022-06-28-YY0126/Concise Summary Report (60)_files/score_gifLMAWMBUQ.gif]

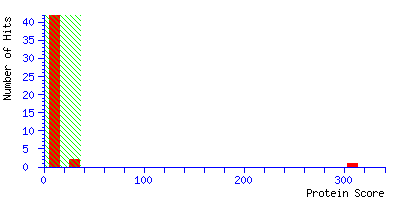

Supplement: Supplementary file 4 — Source Data [file 41467_2022_33690_MOESM4_ESM.zip › TOF sourcedata/2022-06-28-YY0126/Concise Summary Report (80)_files/score_gifNYQZPD0T.gif]
